# Ultrasound Enthesitis in Psoriasis Patients with or without Psoriatic Arthritis, a Cross-Sectional Analysis

**DOI:** 10.3390/medicina58111557

**Published:** 2022-10-30

**Authors:** Mihaela Agache, Claudiu C. Popescu, Liliana Popa, Cătălin Codreanu

**Affiliations:** 1 Department of Internal Medicine and Rheumatology, “Carol Davila” University of Medicine and Pharmacy, 050474 Bucharest, Romania; 2 Clinical Center of Rheumatic Diseases, 030167 Bucharest, Romania; 3 Department of Dermatology and Allergology, Elias Emergency University Hospital, 011461 Bucharest, Romania

**Keywords:** psoriasis, psoriatic arthritis, enthesitis, ultrasound

## Abstract

*Background and objectives:* The main objective of the current study was to describe the prevalence of enthesitis at different sites in a group of patients with psoriasis with or without psoriatic arthritis (PsA). *Materials and Methods:* The study included adult patients with psoriasis who underwent clinical examination, laboratory tests and ultrasound examination of the entheses. The enthesitis ultrasound scores (BUSES, MASEI, GUESS) were evaluated; the presence of OMERACT-defined enthesitis was also recorded for each scan site. *Results*: The study included 16 (57.1%) patients with PsA and 12 (42.9%) patients with psoriasis, with an increased average body mass index (29.3 kg/m^2^). Compared to psoriasis patients, PsA patients had a higher prevalence of nail psoriasis (68.8% compared to 33.3%; *p* = 0.063). There were no significant differences regarding the clinical examination of entheses between patients with psoriasis and patients with PsA (*p* = 0.459). Ultrasound scores, BUSES, GUESS and MASEI proved to have statistically significant higher median values in PsA patients compared to psoriasis patients. Compared to psoriasis patients, PsA patients had a significantly higher prevalence of OMERACT-defined enthesitis of the quadriceps tendon and inferior patellar ligament (both 81.3% compared to 25.0%, *p* = 0.003). Clinical examination of the lateral epicondyle and of the superior patellar ligament was consistent with their ultrasound examination (κ = 0.357, *p* = 0.043, respectively, κ = 0.404, *p* = 0.008). *Conclusions*: Clinical enthesitis scores do not differ between psoriasis and PsA patients. All analyzed ultrasound scores are significantly higher in patients with PsA. OMERACT-defined enthesitis has the ability to discriminate sonographic enthesitis between the two subgroups for bilateral quadriceps and inferior patellar tendon enthesitis. Bilateral ultrasound damage of entheses can suggest a PsA diagnosis.

## 1. Introduction

Enthesitis is defined as the inflammation of the insertion of a tendon, ligament, or capsule at the adjacent bone. It represents one of the characteristics of psoriatic arthritis (PsA) having diagnostic and prognostic value [1,2]. Both the European Alliance of Associations for Rheumatology (EULAR) and the Group for Research and Assessment of Psoriasis and Psoriatic Arthritis (GRAPPA) dedicated a special treatment chapter to enthesitis [3,4]. In patients with PsA, enthesitis may occur independently of arthritis [3]. It is proposed as a trigger mechanism in patients with spondyloarthritis, a group of diseases that also includes PsA [5,6]. In 2001, Benjamin and McGonagle proposed, through a histological description, the concept of “enthesis organ” [5]: the tendon/ligament insertion, as well as the fibrocartilage, fat pad and adjacent bursa, all contributing to the energy dissipation in the bone through entheses.

The gold standard in the assessment of entheses is the histological examination, but this cannot be conducted for ethical reasons and practical limitations. In clinical practice, entheses are assessed by clinical and imaging examination (ultrasound, magnetic resonance imaging—MRI). The clinical examination of entheses relies on assessing tenderness [7], namely by applying a digital pressure of 4 kg/cm^2^ at the insertion sites.

Among the imaging methods used, musculoskeletal ultrasound is in the first line, being currently a cheap, reproducible, non-irradiating method, easily accepted by the patient. After 20 years from the first description of enthesitis in 1994 by Lehtinen et al. [8], the Outcome Measures in Rheumatology (OMERACT) group developed, by consensus with a Delphi exercise in 2014 [9], the definition of the core components of an enthesitis in order to standardize clinical research. Later OMERACT validated the definition of enthesitis in 2019 [10,11], considering a hypoechoic and/or thickened insertion at a distance of at least 2 mm from the bone that may or may not present power Doppler (PD) signal as a positive diagnosis; other elements, such as enthesophites, calcifications or erosions are also considered. Regarding interobserver-agreement for ultrasound examination of enthesitis, the OMERACT group reported among 11 experienced rheumatologists a prevalence of 60% and a bias-adjusted kappa of 0.6 [10], with an interval between 10% for enthesial thickening and 70% for osteophytes. Using the same OMERACT definition, Di Matteo et al. developed a multicenter reliability study [12] with similar results. On the other hand, Di Mateo et al. [13] identified changes in 34.1% of 82 healthy volunteers at the level of entheses (28.0% presented an increase in enthesis thickness, 13.4% hypoechogenicity and 9.8% PD signals), emphasizing the importance of developing a score that distinguishes between enthesitis associated with inflammatory or non-inflammatory diseases. Of all the core components defined by OMRACT, it is well known that the PD signal is more sensitive in detecting early enthesitis, being considered its most diagnostic feature [13,14,15].

Epidemiologically, PsA is a particular disease since patients are individualized from a certain group of subjects, respectively, 6% to 41% of patients with psoriasis will develop PsA [16]. There are studies that aimed to characterize a predictive model of PsA development in patients with psoriasis, both by clinical evaluation of the entheses (clinical enthesopathy predicted the occurrence of PsA) [17] and also by ultrasound [18]. In this latter predictive model, quadriceps and patellar enthesitis had a risk ratio of 1.96 (*p* < 0.005) in predicting the development of PsA.

In this context, the main objective of the current study was to describe the prevalence of enthesitis on various sites in a group of patients with psoriasis with or without PsA and to evaluate the discriminative capacity of ultrasonographic enthesitis sites, in order to possibly identify specific entheses sites and their ultrasound modifications that can differentiate the two conditions.

## 2. Materials and Methods

### 2.1. Patients

The study included all adult psoriasis patients with or without PsA, according to CASPAR criteria [19], who presented to the outpatient clinic of a tertiary rheumatology university hospital between February and July 2022 and who agreed to participate. Psoriasis patients were referred by local dermatology services which diagnosed them with the dermatological disease. On the same day, each patient underwent clinical examination, laboratory tests and ultrasound examination.

The study was carried out in accordance with the standards of the Declaration of Helsinki, with the approval of the Ethics Committee of the institution and each patient signed informed consent before the study.

### 2.2. Demographic Characteristics, Clinical and Laboratory Tests

Blinded to imaging studies, a senior rheumatologist performed comprehensive medical history and clinical examination of all patients, including tender joints count (TJC) and swollen joints count (SJC) from 66/68 joints [20], resulting in the composite disease activity score DAPSA (Disease Activity Index for Psoriatic Arthritis) [21]. Clinical examination also included enthesis, resulting in the Spondyloarthritis Research Consortium of Canada (SPARCC) score with 16 entheses (bilateral greater trochanter, quadriceps tendon insertion into the patella, patellar ligament insertion into the patella and tibial tuberosity, Achilles tendon insertion, plantar fascia insertion, medial and lateral epicondyles, and supraspinatus insertion) [22].

Patient age was calculated as the difference between the study inclusion date and birthdate. Body mass index (BMI) was calculated as the ratio of weight to squared height, and obesity was defined as BMI ≥ 30 kg/m^2^. Retrospectively, from the patient’s medical history, dyslipidemia was defined as either total serum cholesterol or triglycerides over the upper limit of normal according to the respective local laboratory or if the patient was under current lipid-lowering treatment (statins, fibrates). Metabolic syndrome was defined as obesity with at least two of arterial hypertension, diabetes mellitus and dyslipidemia.

From the peripheral venipuncture blood samples, the laboratory determined for each patient acute phase reactants (C-reactive protein, CRP, with an upper limit of normal, ULN, of 5 mg/L, and erythrocyte sedimentation rate, ESR, with a ULN of 20 mm/h), serum uric acid (ULN of 6.3 mg/dL) and immunological markers (rheumatoid factor, RF, with a ULN of 30 IU/mL and anti-citrullinated protein antibodies, ACPA, with a ULN of 15 IU/mL).

### 2.3. Ultrasound Examination

Ultrasound examinations were performed with an Esaote MyLab Twice machine, using a linear probe with a frequency of 6–18 Mhz. The evaluation was performed in grayscale (GS) and PD with a pulse repetition frequency (PRF) of 750 MHz and a low filter. Scans were performed by a certified rheumatologist with more than 5 years of experience in musculoskeletal ultrasound, blinded to the clinical examination, who interpreted the images while they were acquired. The position of the patient’s joints was according to the EULAR recommendations, with the tendon stretched to avoid anisotropy and unflexed to evaluate PD signals [23].

**Figure 1:**
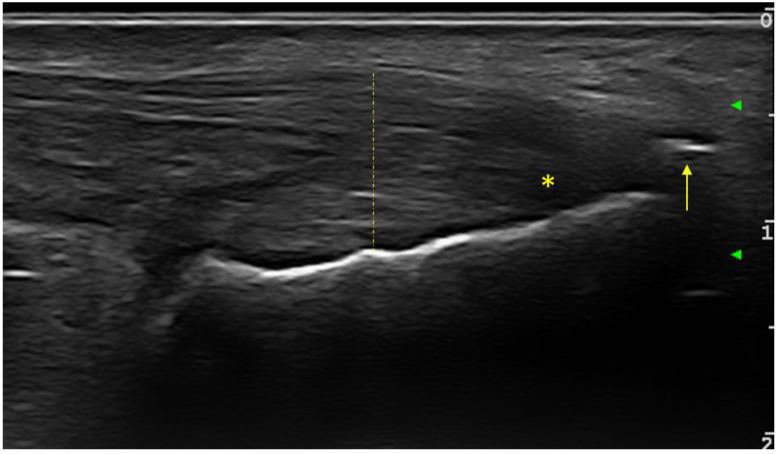
Longitudinal ultrasound scan of Achilles tendon OMERACT-defined enthesitis at calcaneus insertion in a PsA patient (grayscale examination): hypoechoic aspect (star), thickness increase (dashed line) and the presence of enthesophytes (arrow).

The following entheses were evaluated bilaterally in transverse and longitudinal sections: supraspinatus tendons, common extensor digitorum tendon, common digitorum flexor tendon, triceps tendon, quadriceps tendon, proximal and distal patellar tendon, Achilles tendon and plantar fascia. At each individual enthesis, the presence or absence of the following elements was evaluated: thickening, hypoechogenicity, calcifications/enthesophytes, erosions and the PD signal. The evaluated entheses allowed for the calculation of the Glasgow Ultrasound Enthesitis Scoring System (GUESS) [24], Madrid Sonographic Enthesitis Index (MASEI) [25] and Belgrade Ultrasound Enthesitis Score (BUSES) [26] sonographic enthesitis scores, in order to assess the discriminative capacity between the two groups of patients. For all examined entheses (upper and lower limb), the presence or absence of any of the evaluated changes (hypoechogneicity, increased thickness, calcifications/enthesitis, bursitis, or PD signal) was bilaterally noted. At the level of the lower limb, the elementary changes in the OMERACT definition of enthesitis were included (Figure 1).

### 2.4. Statistics

Data distribution normality was assessed using descriptive statistics, normality, stem-and-leaf plots and the Lillefors-corrected Kolmogorov–Smirnov tests. Continuous variables were reported as “mean ± standard deviation” if normally distributed, or as “median (minimum-maximum)” if non-normally distributed, while dichotomous variables were reported as “observed frequency (percentage of subgroup)”.

Mann–Whitney U tests were used to assess differences in continuous variables among subgroups of dichotomous categorical variables, while the associations between categorical variables were studied using χ^2^ or Fisher’s exact tests.

The agreement of clinical and ultrasound examinations was evaluated with overall agreement, positive agreement, Cohen’s κ (kappa; strength of agreement: κ < 0.2 poor, κ = 0.21–0.40 fair, κ = 0.41–0.60 moderate, κ = 0.61–0.80 good and κ > 0.80 very good) [27]; sensitivity, specificity and positive likelihood ratio (PLR; effect on increasing probability of involvement detection: PLR > 10 large, PLR = 5–10 moderate, PLR < 5 small) [28].

The statistical tests were considered significant if *p* < 0.05. The statistical analysis was performed using IBM SPSS Statistics version 25.0 for Windows (IBM Corp., Armonk, New York, USA).

## 3. Results

### 3.1. Demographic Characteristics of the Patients

The study included 28 patients, respectively, 16 (57.1%) patients with PsA and 12 (42.9%) patients with skin psoriasis, of which 71.4% were women, with an average age of 54.7 years (Table 1). Compared to psoriasis patients, those with PsA had a significantly higher median age (59.7 compared to 48.2 years; *p* = 0.044), a significantly higher prevalence of arterial hypertension (62.5% compared to 16.7%; *p* = 0.015) and a higher prevalence of metabolic syndrome (25.0% compared to none; *p* = 0.061). These characteristics can be explained by the fact that the transition from skin psoriasis to psoriatic arthritis is a continuum process, a period of time in which the patient continues to accumulate comorbidities. Of note, the study sample had an increased average BMI (29.3 kg/m^2^), without a significant difference between subgroups. There were no differences between subgroups related to smoking and manual labor.

### 3.2. Psoriasis and PsA Characteristics

Compared to patients with psoriasis, patients with PsA had a higher prevalence of nail psoriasis (68.8% compared to 33.3%; *p* = 0.063) and of treatment with conventional synthetic disease-modifying antirheumatic drugs (csDMARDs; 68.8% compared to 33.3%; *p* = 0.063), significantly higher median SJC (1 compared to 0; *p* = 0.039) and inflammatory markers (CRP: 9.4 mg/L compared to 1.5 mg/L, *p* = 0.044, and ESR: 23 mm/h compared to 6 mm/h, *p* = 0.006; Table 2).

Regarding PsA activity measured by DAPSA score, 1 (6.3%) patient was in remission, 2 patients (12.5%) had low disease activity, 11 patients (68.8%) had moderate disease activity and 2 (12.5%) patients had high disease activity. The was only one patient with positive RF (3.6%) and another patient with positive ACPA (3.6%).

### 3.3. Clinical Examination of Entheses

There were no significant differences regarding the clinical examination of entheses between patients with psoriasis and patients with PsA, both for the frequency of positive sites (Table 3) or for SPARCC score, which had a median value of 6 (0–22) in psoriasis patients and 7 (0–27) in PsA patients (*p* = 0.459). Compared to non-obese patients, obese patients had a significantly higher prevalence of painful plantar fascia (50.0% compared to 12.5%, *p* = 0.030).

### 3.4. Ultrasound Examination of Entheses

Ultrasound examination of the quadriceps tendon insertion on the superior pole of the patella revealed a significant difference, namely the lower prevalence of normal ultrasound (25.0% compared to 50.0%; *p* = 0.032) and the higher prevalence of bilateral involvement in PsA compared to psoriasis patients (56.3% compared to 8.3%; *p* = 0.032; Table 4).

Compared to patients without metabolic syndrome, patients with metabolic syndrome had a significantly higher prevalence of positive ultrasound findings in triceps tendon insertion on the olecranon (50.0% compared to 4.2%, *p* = 0.045).

The ultrasound scores BUSES, GUESS and MASEI proved to have statistically significant higher median values in PsA patients compared to psoriasis patients (Table 5). Compared to psoriasis patients, PsA patients had a significantly higher prevalence of OMERACT-defined enthesitis of the quadriceps tendon and inferior patellar ligament (both 81.3% compared to 25.0%, *p* = 0.003, Table 6). Part of these differences is explained by the significantly higher prevalence of bilateral enthesitis at these sites in PsA patients (Table 6). Additionally, PsA patients had a significantly higher prevalence of bilateral OMERACT-defined enthesitis of the superior patellar ligament (37.5% compared to none, *p* = 0.039).

The presence of PD signal was rare, being highlighted in 5 out of the 504 evaluated entheses (0.99%), respectively, in two patients with PsA and two patients with psoriasis; four of the five entheses were at the insertion of the common extensor digitorum on the lateral epicondyle, and the fifth at the insertion of the inferior patellar tendon on the tibial tubercle.

### 3.5. Agreement of Clinical and Ultrasound Examination

Clinical examination of the lateral epicondyle and of the superior patellar ligament was consistent with their ultrasound examination (κ = 0.357, *p* = 0.043 and, respectively, κ = 0.404, *p* = 0.008; Table 7).

## 4. Discussion

In summary, the most important results of the study were the lack of correlation between the clinical evaluation of entheses and their ultrasonographic appearance; the degree of enthesitis evaluated by the ultrasound scores that can complement and reinforce each other in differentiating between patients with psoriasis and PsA and the fact that several entheses have been found which can indicate PsA damage rather than psoriasis if bilateral ultrasound changes are present.

Enthesitis in PsA has an important impact, being associated both with ultrasound-detected erosions [29] and with radiological progression [30]. Enthesitis has gained importance in recent years and in clinical trials; therefore, its assessment is useful although the method by which this is conducted is not fully established.

In the current study, we performed a clinical and ultrasound evaluation of enthesitis, in two groups of patients: with psoriasis and PsA. There were no gender-related differences between the analyzed groups. The significantly older age of patients with APs can be explained by the transition from psoriasis to PsA. It is worth noting that patients had an increased BMI in both groups (with an average value of 30.0 kg/m^2^ for PsA patients and 28.3 kg/m^2^ for psoriasis patients). Additionally, the higher prevalence of nail involvement in PsA patients reinforces that psoriatic nail is a predictive factor for the progression from psoriasis to PsA [18,31].

For the clinical examination, we chose the SPARCC score because there is a large overlap of the sites evaluated by the ultrasound scores. No statistically significant differences were detected between patients with PsA and psoriasis both for the frequency of positive sites or for SPARCC scores. This can be explained by the existence of a cofounder or by the fact that patients with PsA are not naïve to treatment, their medication may underestimate the clinical prevalence of enthesitis. Pain on applied pressure is not the best indicator of enthesitis.

The enthesitis sites scanned in this study were chosen according to a recent GRAPPA publication [32], where the sensitivity and specificity of each site are specified. Ultrasound examination of the quadriceps tendon insertion on the superior pole of the patella revealed a significant difference: a high prevalence of bilateral involvement in PsA compared to psoriasis patients.

The recently published ULISSE study compares the prevalence of clinical and ultrasonographic enthesal involvement in PsA, psoriasis and fibromyalgia patients [33]. Patients with PsA and psoriasis had a comparable number of entheses with at least one ultrasound change, the only relevant differences were in the Achilles tendon and quadriceps tendon entheses affected in pairs in patients with PsA.

Related to the agreement between the clinical examination and ultrasonography for each site, an agreement between the clinical examination and any of the ultrasound signs (any lesion including hypoechogenicity, thickening, calcifications, enthesitis, bursitis, PD signal) of enthesitis was calculated. This study highlighted an acceptable agreement only at the level of the lateral epicondyle (κ = 0.357, *p* = 0.043) and superior patellar tendon (κ = 0.404, *p* = 0.008). Most studies in the literature have reported discordance between clinical and sonographic evaluations [32,34], but the methods by which this comparison is made differ (comparison of scores or enthesitis sites).

Kristensen et al. [35] show a moderate correlation between the ultrasound elements of enthesitis and the LEI and SPARCC clinical scores (r = 0.50, respectively r = 0.47). The correlation is stronger between hypoechogenicity and the increased thickness of enthesis and LEI and SPARCC (respectively, r = 0.81 and r = 0.86).

It is known that the tenderness expressed by clinical evaluation can interfere with the adjacent tendons and joints with the pain threshold, or it can be generated by the osteitis adjacent to the insertion. The clinical evaluation of entheses can overlap with the trigger points from other central hypersensitivity syndromes. Patients with fibromyalgia have diffuse pain including entheses, causing clinically higher enthesitis scores without differences in the inflammatory ultrasound scores [33]. The fact that studies show that the prevalence of pain at entheses points is higher in patients with fibromyalgia than in patients with PsA or psoriasis demonstrates that pain on digital pressure is not a specific indicator for enthesitis. Many entheses points near joints are fibromyalgia trigger points and they can be misinterpreted [36,37,38,39,40].

There are also non-inflammatory enthesopathies, especially of the lower limbs, which appear in case of an increased BMI, older age, overuse of certain joints (for example, jumper’s knee of young athletes [41]), or in the presence of metabolic syndrome [42,43,44]. In the present study, compared to non-obese patients, obese patients had a significantly higher prevalence of painful plantar fascia (50.0% compared to 12.5%, *p* = 0.030).

The ultrasound scores BUSES, MASEI and GUESS were significantly higher among patients with PsA compared to psoriasis patients.

The BUSES [26] score does not differentiate between inflammatory and non-inflammatory changes but the MASEI and GUESS [24,25] scores take into account the presence or absence of PD signals. Eder et al. [45] detected the following MASEI scores in patients with PsA, psoriasis and healthy controls, respectively, 13, 6 and 3.5. Additionally, the same study reported the presence of PD signals in 8.3% of healthy subjects. Polaceck et al. demonstrated that the MASEI ultrasound score correlates with radiological progression [46]. A further study direction would be to establish cut-off values for these indices, which could facilitate the moment of diagnosis in the transition from psoriasis to PsA.

According to the latest OMERACT [11] and EFSUMB [15] recommendations, the presence of a PD signal is the most sensitive tool in the diagnosis of enthesitis that differentiates an active enthesis from an inactive one. The percentage of PD was low in our study (0.99%), most often at the level of the lateral epicondyle. Additionally, in a study by Kristensen et al., a PD signal was detected at the level of entheses in only a few patients [35]. Similarly, a slightly higher percentage was detected by Sapsford et al. in patients with PsA—respectively, 3.4% of total sites, most frequently at the level of the lateral humeral epicondyle, quadriceps tendon and inferior patellar insertion [40]. The same study, also using the OMERACT definition, reported that the most frequent ultrasound-affected entheses are the insertion of the quadriceps and the Achilles tendon. In our study four of the five entheses with PD signals were at the insertion of the common extensor digitorum on the lateral epicondyle, which is an area of recent interest for researchers who have even focused on its histological study [47].

Although it included a small sample of patients, the present study, as far as we know, is one of the few studies that evaluate the clinical and sonographic findings of enthesitis in patients with PsA versus patients with psoriasis. Other limitations of the study are the facts that the entheses were evaluated by only one sonographer; it was not possible to calculate cut-offs for enthesitis scores that could differentiate patients with PsA from patients with psoriasis (as other authors have reported [48]); the use of systemic therapy in PsA may have suppressed the possibility of detecting PD signals. Even though there is no agreement on the optimal number of entheses and the specific sites at which to perform the ultrasound evaluation, entheses remain routinely analyzed in clinical trials to evaluate the effectiveness of treatments.

## 5. Conclusions

The clinical scores of enthesitis do not differ between the groups of patients with psoriasis and PsA. All analyzed ultrasound scores (BUSES, MASEI, GUESS) are significantly higher in PsA patients. OMERACT-defined enthesitis has a discriminative capacity of sonographic enthesitis sites between the two subgroups for the bilateral enthesitis of the quadriceps and inferior patellar tendon. Bilateral ultrasound damage of entheses can suggest a PsA diagnosis. These findings reiterate the need for clinical trials in PsA to use ultrasound evaluation for enthesitis assessment.

## Figures and Tables

**Table 1:** Demographics and general characteristics of patients.

	All (*n* = 28)	Pso (*n* = 12)	PsA (*n* = 16)	*p*
Age (y)	54.7 ± 15.3	48.2 ± 12.8	59.7 ± 15.5	0.044
Women	71.4%	66.7%	75.0%	0.629
Smoking	42.9%	41.7%	43.8%	0.912
BMI (kg/m^2^)	29.3 ± 5.1	28.3 ± 6.7	30.0 ± 3.5	0.252
Obesity	42.9%	25.0%	56.3%	0.098
Manual labor	32.1%	33.3%	31.3%	0.907
AHT	42.9%	16.7%	62.5%	0.015
Dyslipidemia	35.7%	33.3%	37.5%	0.820
DM	10.7%	0	18.8%	0.238
MetS	14.3%	0	25.0%	0.061

AHT—arterial hypertension; BMI—body mass index; DM—type 2 diabetes mellitus; MetS—metabolic syndrome; PsA—psoriatic arthritis; Pso—psoriasis; SD—standard deviation; y—years.

**Table 2:** General characteristics of psoriasis and PsA patients.

	All (*n* = 28)	Pso (*n* = 12)	PsA (*n* = 16)	*p*
Age of Pso onset (y)	36.8 ± 15.3	35.8 ± 13.3	38.4 ± 17.2	0.900
Scalp Pso	67.9%	58.3%	75.0%	0.350
Nail Pso	53.6%	33.3%	68.8%	0.063
Dactylitis	35.7%	33.3%	37.5%	0.820
Arthralgia	78.6%	75.0%	81.3%	0.690
Age of arthralgia onset (y)	50.5 ± 14.7	42.3 ± 10.7	53.7 ± 15.1	0.080
TJC	7 (0–24)	3 (0–16)	8 (1–24)	0.276
SJC	0 (0–5)	0 (0–1)	1 (0–4)	0.039
CRP (mg/L)	5.2 (0.7–41.7)	1.5 (0.2–41.7)	9.4 (1.1–34.4)	0.044
ESR (mm/h)	15 (2–51)	6 (2–38)	23 (3–51)	0.006
SUA (mg/dL)	4.7 (3.0–9.2)	4.8 (3.0–6.1)	4.9 (3.5–9.2)	0.900
csDMARD	53.6%	33.3%	68.8%	0.063
bDMARD	17.9%	16.7%	18.8%	0.887
Age at PsA diagnosis (y)	-	-	54.0 (16.3)	-
DAPSA	-	-	18.8 (8.8)	-

CRP—C-reactive protein; b/csDMARD—biologic or conventional synthetic disease-modifying antirheumatic drug; DAPSA—Disease Activity in Psoriatic Arthritis; ESR—erythrocyte sedimentation rate; PsA—psoriatic arthritis; Pso—psoriasis; SD—standard deviation; SJC—swollen joint count; SUA—serum uric acid; TJC—tender joint count; y—years.

**Table 3:** Clinical examination of entheses.

	No Involvement			Unilateral Involvement			Bilateral Involvement			*p*
	All	Pso	PsA	All	Pso	PsA	All	Pso	PsA	
ST	67.9%	83.3%	56.3%	17.9%	16.7%	18.8%	14.3%	0	25.0%	0.153
ME	32.1%	33.3%	31.3%	32.1%	25.0%	37.5%	35.7%	41.7%	31.3%	0.759
LE	50.0%	41.7%	56.3%	25.0%	25.0%	25.0%	25.0%	33.3%	18.8%	0.646
TT	85.7%	83.3%	87.5%	7.1%	8.3%	6.3%	7.1%	8.3%	6.3%	0.953
QT	64.3%	75.0%	56.3%	25.0%	16.7%	31.3%	10.7%	8.3%	12.5%	0.586
PL	67.9%	58.3%	75.0%	14.3%	25.0%	6.3%	17.9%	16.7%	18.8%	0.371
AT	71.4%	75.0%	68.8%	14.3%	16.7%	12.5%	14.3%	8.3%	18.8%	0.726
PF	71.4%	83.3%	62.5%	25.0%	16.7%	31.3%	3.6%	0	6.3%	0.417

Pso (*n* = 12 patients) and PsA (*n* = 16 patients); AT—Achilles tendon insertions into the calcaneus; LE—common extensor tendon insertion into the lateral epicondyle; ME—common flexor tendon insertion into the medial epicondyle; PF—plantar fascia insertions into the calcaneus; PL—patellar ligament insertions into the patellar apex and tibial tuberosity; QT—quadriceps tendon insertion on the superior pole of the patella; ST—supraspinatus tendon insertion into the superior facet of the humerus; TT—triceps tendon insertion on the olecranon; other: Pso—psoriasis; PsA—psoriatic arthritis.

**Table 4:** Ultrasound examination of entheses.

	No Involvement			Unilateral Involvement			Bilateral Involvement			*p*
	All	Pso	PsA	All	Pso	PsA	All	Pso	PsA	
ST	57.1%	58.3%	56.3%	28.6%	41.7%	18.8%	14.3%	0	25.0%	0.119
ME	92.9%	83.3%	100%	7.1%	16.7%	0	0	0	0	0.175
LE	39.3%	58.3%	25.0%	46.4%	25.0%	62.5%	14.3%	16.7%	12.5%	0.129
TT	89.3%	91.7%	87.5%	7.1%	8.3%	6.3%	3.6%	0	6.3%	0.669
QT	35.7%	50.0%	25.0%	28.6%	41.7%	18.8%	35.7%	8.3%	56.3%	0.032
sPL	89.3%	83.3%	93.8%	10.7%	16.7%	6.3%	0	0	0	0.378
iPL	89.3%	91.7%	87.5%	10.7%	8.3%	12.5%	0	0	0	0.724
AT	60.7%	66.7%	56.3%	17.9%	16.7%	18.8%	21.4%	16.7%	25.0%	0.835
PF	71.4%	83.3%	62.5%	17.9%	16.7%	18.8%	10.7%	0	18.8%	0.261

Pso (*n* = 12 patients) and PsA (*n* = 16 patients). AT—Achilles tendon insertions into the calcaneus; LE—common extensor tendon insertion into the lateral epicondyle; ME—common flexor tendon insertion into the medial epicondyle; PF—plantar fascia insertions into the calcaneus; PL—superior/inferior patellar ligament insertions into the patellar apex and tibial tuberosity; QT—quadriceps tendon insertion on the superior pole of the patella; ST—supraspinatus tendon insertion into the superior facet of the humerus; TT—triceps tendon insertion on the olecranon; other: Pso—psoriasis; PsA—psoriatic arthritis.

**Table 5:** Comparison of enthesitis indices.

		All	Pso	APs	*p*
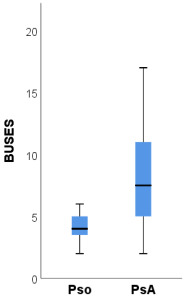	BUSES	6 (2–22)	4 (2–9)	8 (2–22)	0.006
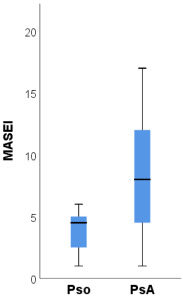	MASEI	6 (1–17)	5 (1–6)	8 (1–17)	0.006
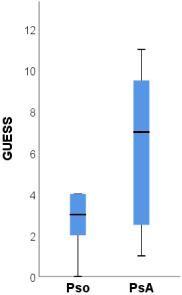	GUESS	4 (0–11)	3 (0–4)	7 (1–11)	<0.001

Pso (*n* = 12 patients) and PsA (*n* = 16 patients). BUSES—Belgrade Ultrasound Enthesitis Score; GUESS—Glasgow Ultrasound Enthesitis Scoring System; MASEI—Madrid Sonographic Enthesitis Index; Pso—psoriasis; PsA—psoriatic arthritis; SPARCC—The Spondyloarthritis Research Consortium of Canada Enthesitis Index.

**Table 6:** OMERACT-defined ethesitis in the lower limb in diagnostic subgroups.

	Any			No		Unilateral		Bilateral		*p*
	Pso	PsA	p	Pso	PsA	Pso	PsA	Pso	PsA	
QT	25.0%	81.3%	0.003	75.0%	18.8%	25.0%	43.8%	0	37.5%	0.006
sPL	41.7%	75.0%	0.074	58.3%	25.0%	41.7%	37.5%	0	37.5%	0.039
iPL	25.0%	81.3%	0.003	75.0%	18.8%	25.0%	25.0%	0	56.3%	0.003
AT	8.3%	31.3%	0.144	91.7%	68.8%	0	31.3%	8.3%	0	0.063
PF	25.0%	43.8%	0.306	75.0%	56.3%	25.0%	12.5%	0	31.3%	0.094

Pso (*n* = 12 patients) and PsA (*n* = 16 patients). AT—Achilles tendon insertions into the calcaneus; QT—quadriceps tendon insertion on the superior pole of the patella; PF—plantar fascia insertions into the calcaneus; PL—superior/inferior patellar ligament insertions into the patellar apex and tibial tuberosity; other: OMERACT—Outcome Measure in Rheumatology; Pso—psoriasis; PsA—psoriatic arthritis.

**Table 7:** Agreement of clinical and ultrasound examination (*n* = 28).

	*κ*	*p*	OA	PA	Se	Sp	PLR
ST	0.022	0.907	53.6%	44.4%	33.3%	68.8%	1.1
ME	0.070	0.312	39.3%	10.5%	100.0%	34.6%	1.5
LE	0.357	0.043	67.9%	78.6%	64.7%	72.7%	2.4
TT	0.186	0.318	82.1%	25.0%	33.3%	88.0%	2.8
QT	0.075	0.638	50.0%	70.0%	38.9%	70.0%	1.3
PL	0.404	0.008	78.6%	33.3%	100.0%	76.0%	4.2
AT	0.135	0.463	60.7%	50.0%	36.4%	76.5%	1.5
PF	0.125	0.508	64.3%	37.5%	37.5%	75.0%	1.3

AT—Achilles tendon insertions into the calcaneus; LE—common extensor tendon insertion into the lateral epicondyle; ME—common flexor tendon insertion into the medial epicondyle; PF—plantar fascia insertions into the calcaneus; PL—superior/inferior patellar ligament insertions into the patellar apex and tibial tuberosity; QT—quadriceps tendon insertion on the superior pole of the patella; ST—supraspinatus tendon insertion into the superior facet of the humerus; TT—triceps tendon insertion on the olecranon; other: κ—Cohen’s kappa; OA—overall agreement; PA—positive agreement; PLR—positive likelihood ratio; Se—sensitivity; Sp—specificity.

## Data Availability

The data presented in this study are available on request from the corresponding author. The data are not publicly available due to patient privacy restrictions.
